# Supplementary material for: Exploring the K+ binding site and its coupling to transport in the neurotransmitter:sodium symporter LeuT
Source: eLife. 2024 Jan 25;12:RP87985. doi: 10.7554/eLife.87985 (PMC10945697; doi:10.7554/eLife.87985)
Supplement: Table 1—source data 1. [file elife-87985-table1-data1.docx]

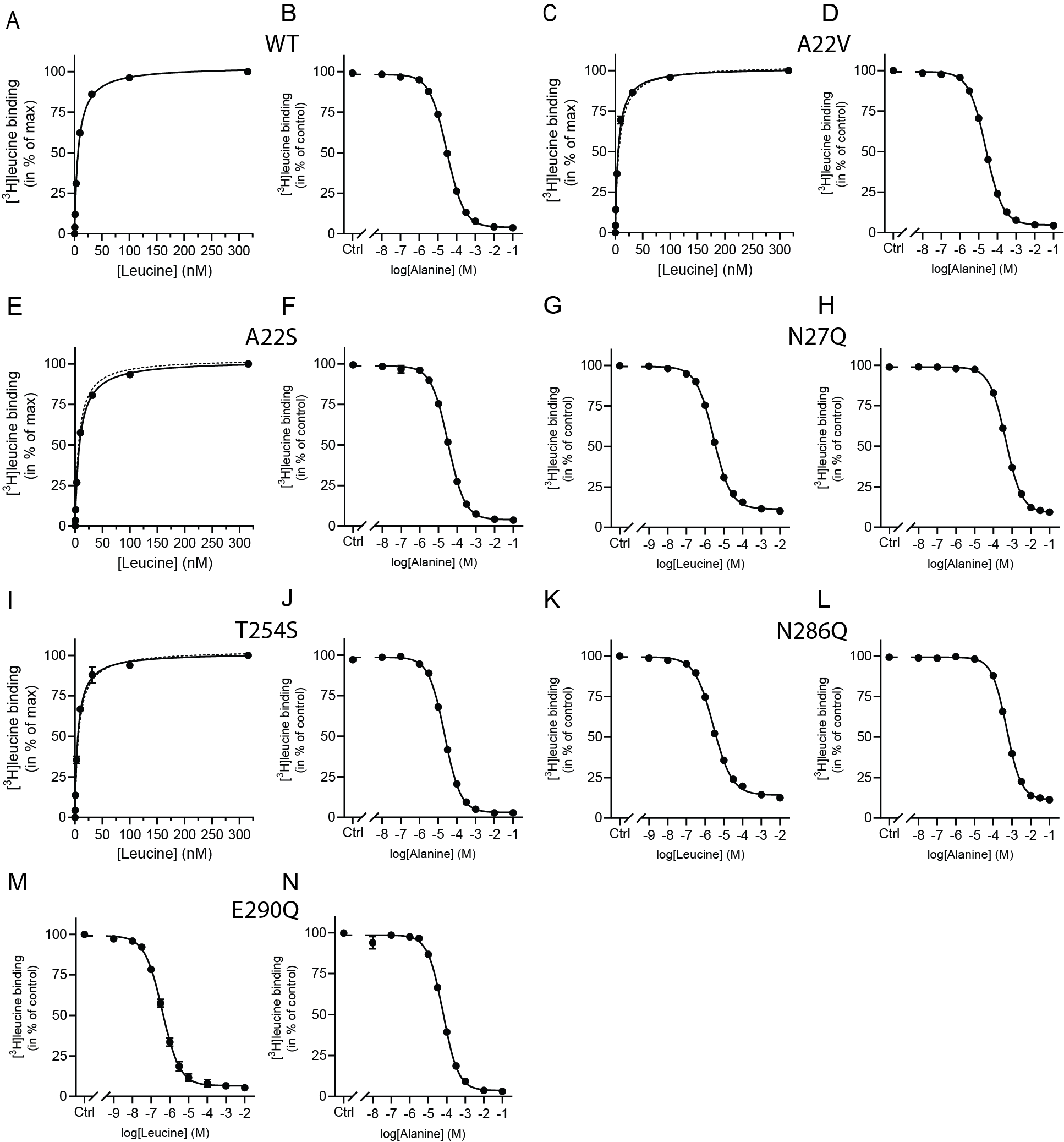


**Table 1 – Source data 1. Affinity curves for leucine and alanine binding to LeuT and mutants. (A**,**C**,**E**,**I)** [^3^H]leucine saturation binding for WT (A), A22V (C), A22S (E) and T254S (I). (**B**,**D**,**F-H,J-N**) Displacement of [^3^Hleucine by leucine for N27Q (G), N286Q (K) and E290Q (M), or by alanine for WT (B), A22V (D), A22S (F), N27Q (H), T254S (J), N286Q (L) and E290Q (N). Binding was assayed in 200 mM Na^+^. All data points are mean ± s.e.m., n = 3-4, and fitted to a one-site binding model (A, C, E, I) or non-linear regression fit. *K*_d_ and *K*_i_ values are summarized in Table 1. All data is provided in the source data file.
