# Supplementary material for: Exploring the K+ binding site and its coupling to transport in the neurotransmitter:sodium symporter LeuT
Source: eLife. 2024 Jan 25;12:RP87985. doi: 10.7554/eLife.87985 (PMC10945697; doi:10.7554/eLife.87985)
Supplement: Figure 3—source data 1. [file elife-87985-fig3-data1.docx]

|  | | | | |
| --- | --- | --- | --- | --- |
| **Intra-vesicular cation** | **Plateau ± s.e.m.**  **(% of uptake in intra-vesicular NMDG^+^)** | **k ± s.e.m.**  **(min^-1^)** | **R^2^** | **N** |
| K^+^ | 255 ± 20 | 0.055 ± 0.011 | 0.85 | 3 |
| Rb^+^ | 254 ± 10 | 0.058 ± 0.006 | 0.96 | 3 |
| Cs^+^ | 93 ± 6 | 0.077 ± 0.015 | 0.80 | 3 |
| NMDG^+^ | 100 ± 3 | 0.056 ± 0.005 | 0.96 | 3 |
| **Figure 3 – Source data 1.**  **Rate constants for time-dependent uptake of [^3^H]alanine by LeuT into proteoliposomes**. Rate constants from time-dependent [^3^H]alanine uptake into proteoliposomes with LeuT fitted to one phase association in GraphPad Prism 9.0 (see Figure 3B). | | | | |
