# Supplementary material for: Exploring the K+ binding site and its coupling to transport in the neurotransmitter:sodium symporter LeuT
Source: eLife. 2024 Jan 25;12:RP87985. doi: 10.7554/eLife.87985 (PMC10945697; doi:10.7554/eLife.87985)
Supplement: Figure 3—source data 2. [file elife-87985-fig3-data2.docx]

|  | | | | |
| --- | --- | --- | --- | --- |
| **Intra-vesicular cation** | ***V*_max_ ± s.e.m.**  **(pmol min^-1^)** | ***K*_m_ ± s.e.m.**  **(mM)** | **R^2^** | **N** |
| K^+^ | 1.93 ± 0.17 | 1.83 ± 0.39 | 0.94 | 3 |
| Rb^+^ | 1.49 ± 0.10 | 1.86 ± 0.28 | 0.97 | 3 |
| Cs^+^ | 0.80 ± 0.11 | 2.36 ± 0.72 | 0.90 | 3 |
| NMDG^+^ | 0.59 ± 0.09 | 1.82 ± 0.70 | 0.83 | 3 |
| **Figure 3 - Supplementary table 2. [^3^H]alanine-dependent uptake by LeuT into proteoliposomes.** Constants from [^3^H]alanine-dependent uptake into proteoliposomes with LeuT containing various intra-vesicular cations fitted to Michaelis-Menten kinetics in GraphPad Prism 9.0 (see Figure 3C). | | | | |
