# Supplementary material for: Exploring the K+ binding site and its coupling to transport in the neurotransmitter:sodium symporter LeuT
Source: eLife. 2024 Jan 25;12:RP87985. doi: 10.7554/eLife.87985 (PMC10945697; doi:10.7554/eLife.87985)
Supplement: Figure 3—source data 3. [file elife-87985-fig3-data3.docx]

|  | | | | |
| --- | --- | --- | --- | --- |
| **Intra-vesicular [K^+^]** | ***V*_max_ ± s.e.m.**  **(% of *V*_max_ in intra-vesicular NMDG^+^)** | ***K*_m_ ± s.e.m.**  **(mM)** | **R^2^** | **N** |
| 0 | 93 ± 20 | 4.07 ± 1.85 | 0.87 | 3 |
| 50 | 118 ± 27 | 3.55 ± 1.85 | 0.83 | 3 |
| 100 | 120 ± 18 | 2.71 ± 1.06 | 0.89 | 3 |
| 150 | 134 ± 12 | 2.64 ± 0.61 | 0.96 | 3 |
| 200 | 179 ± 13 | 1.84 ± 0.40 | 0.96 | 3 |
| **Figure 3 - Supplementary table 3.**  **[^3^H]alanine uptake by LeuT into proteoliposomes with increasing intra-vesicular [K^+^]** Constants from [^3^H]alanine-dependent uptake into proteoliposomes with LeuT containing increasing intra-vesicular [K^+^] fitted to Michaelis-Menten kinetics in GraphPad Prism 9.0 (see Figure 3 – Figure supplement 1B). | | | | |
