# Supplementary figures and images for: Exploring the K+ binding site and its coupling to transport in the neurotransmitter:sodium symporter LeuT
Source: eLife. 2024 Jan 25;12:RP87985. doi: 10.7554/eLife.87985 (PMC10945697; doi:10.7554/eLife.87985)

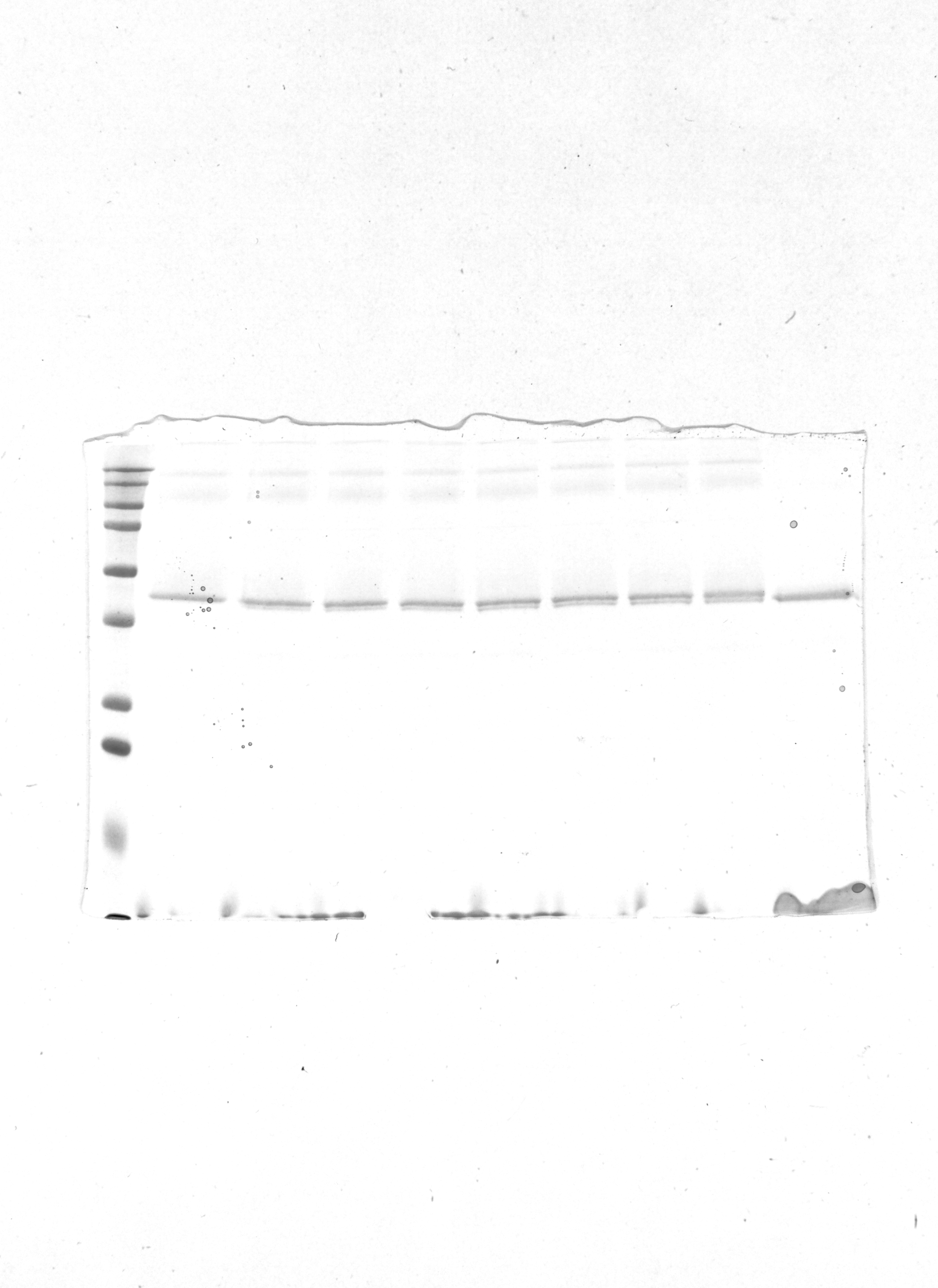

Supplement: Figure 3—figure supplement 1—source data 2. [file elife-87985-fig3-figsupp1-data2.zip › Figure 3-supplementary figure 1-gel unclipped.tif]

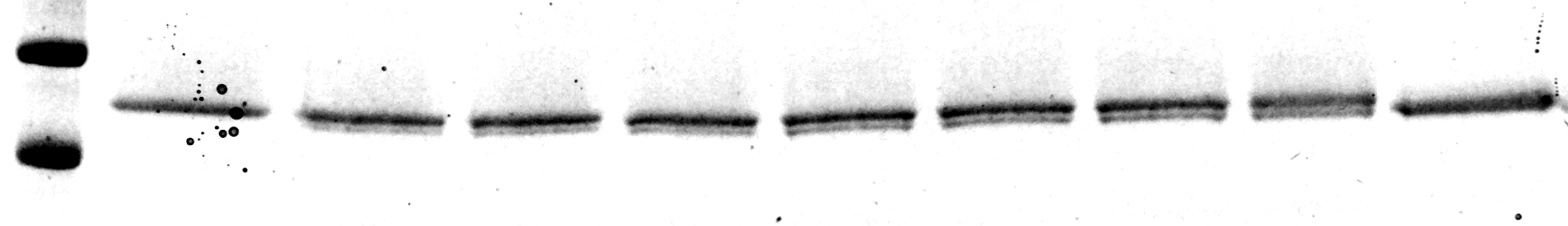

Supplement: Figure 3—figure supplement 1—source data 2. [file elife-87985-fig3-figsupp1-data2.zip › Figure 3-supplementary figure 1-gel.png]
